# Image dataset of tea chrysanthemums in complex outdoor scenes

**DOI:** 10.3389/fpls.2023.1134911

**Published:** 2023-04-14

**Authors:** Siyang Zang, Lei Shu, Kai Huang, Zhiyong Guan, Ru Han, Ravi Valluru, Xiaochan Wang, Jiaxu Bao, Ye Zheng, Yifan Chen

**Affiliations:** ^1^ College of Artificial Intelligence, Nanjing Agricultural University, Nanjing, China; ^2^ School of Engineering, College of Science, University of Lincoln, Lincoln, United Kingdom; ^3^ National Engineering and Technology Center for Information Agriculture (NETCIA), Nanjing Agricultural University, Nanjing, China; ^4^ College of Horticulture, Nanjing Agricultural University, Nanjing, Jiangsu, China; ^5^ Lincoln Institute for Agri-food Technology, University of Lincoln, Lincoln, United Kingdom; ^6^ College of Engineering, Nanjing Agricultural University, Nanjing, Jiangsu, China

**Keywords:** tea chrysanthemum, outdoor scenes, image data, video data, unfixed angle

## 1 Introduction

Chrysanthemums, which originated from China, have an economic value in flower as well as a high value in both edibility and health care in the form of food and tea (Li et al., 2019). With the improved living standards of people, chrysanthemum tea has become a popular drinking target, due to its inherent advantages: 1) it improves the body’s anti-ageing, anti-hypertensive, anti-bacterial, and anti-viral abilities; 2) it regulate the body’s immunity by anti-inflammatory, antipyretic, sedative, and anti-arthritic abilities (Han et al., 2019; Yuan et al., 2019). Hence, the planting area of tea chrysanthemums in China is increasing every year. According to the recent survey (Yang et al., 2018), the planting area in Hangbaiju in Tongxiang City, Zhejiang Province, increased to nearly 4000 hm^2^ with an output of about 12000 kg/hm^2^. Due to its best medicinal properties associated with a specific flowering stage, the harvesting period is very narrow and usually lasts 25 days (Yang et al., 2018). In this regard, there is an urgent need for many farmers to harvest a good quality and quantity of tea chrysanthemums. However, it is difficult to recruit many well-trained farmers in a short time.

During tea chrysanthemums harvesting, the harvesters judge whether to pick or not after observing the state of the tea chrysanthemums flowers. Similarly, the harvestable flowers can be detected by image processing technology, then the picking operation can be completed by the corresponding machines, such as a fruit-picking robot. However, there are many challenges to accurately identifying chrysanthemums in the field due to complex external environmental factors (light, shade, wind, and photo distance, etc.) as well as differences in maturity, colour, and the direction of chrysanthemums’ flower heads. Many researchers have identified chrysanthemums by overcoming some of the above (Yang et al., 2018; Yuan et al., 2018; Liu et al., 2019; Yang et al., 2019; Liu et al., 2020; Qi et al., 2021; Qi et al., 2022a; Qi et al., 2022b), and these studies indicate that:

1) Chrysanthemum flower detection can be realized by machine learning models, which are highly dependent on the quantity and quality of image datasets;
2) The accuracy of flower detection based on RGB images is high, and most of the detection is tested in an ideal environment.

**Figure 1:**
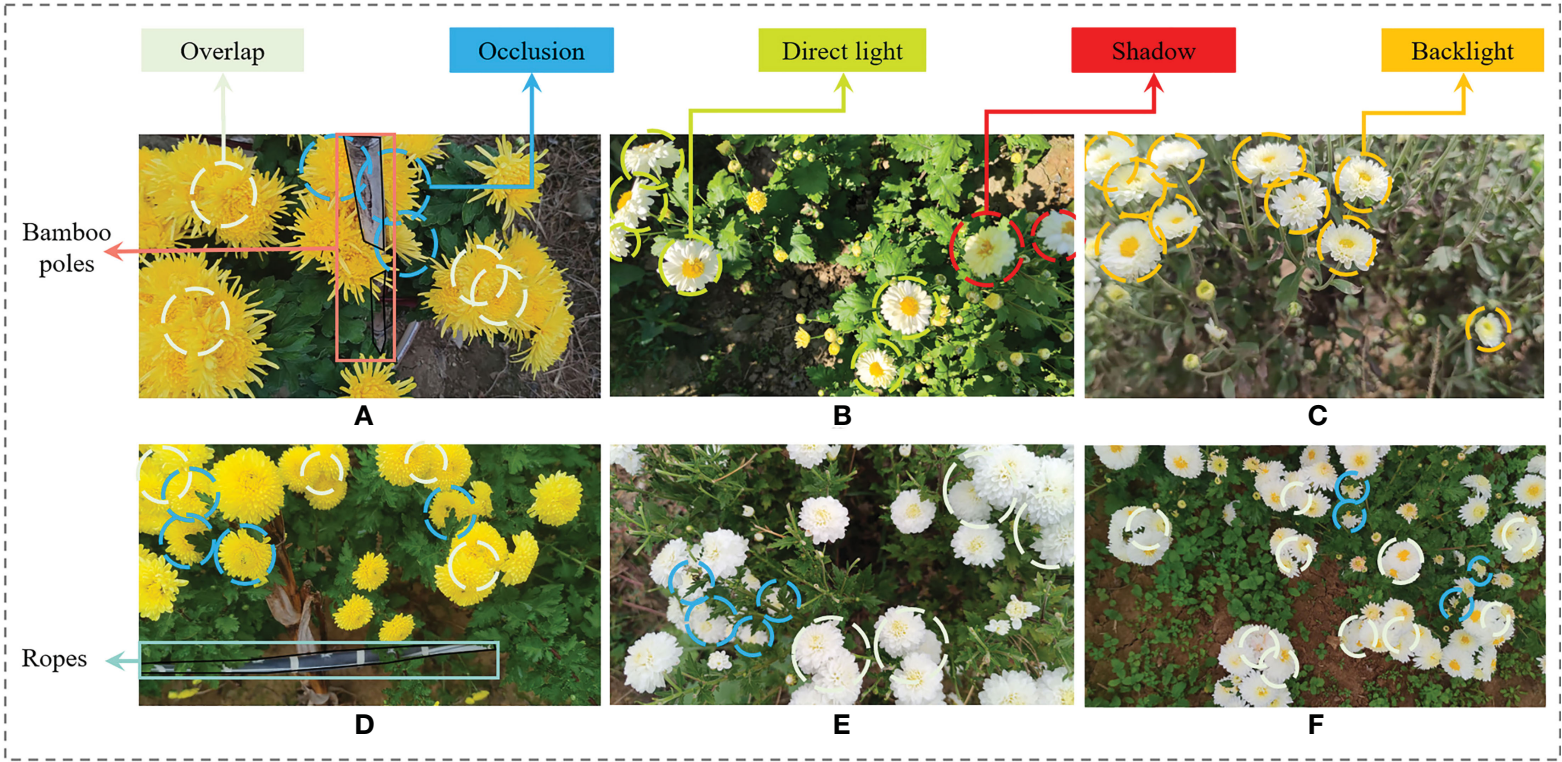
The category to which the sample images belong and their number in the related chrysanthemum dataset: **(A)** *Jinsihuangju*-01314, **(B)** *Hangbaiju*-00063, **(C)** *Bo-chrysanthemum*-11093, **(D)** *Wuyuanhuangju*-03285, **(E)** *Gongju*-00002, and **(F)** *Chuju*-00110.

Due to a large number of varieties of tea chrysanthemums, high planting density, and different field conditions, it is difficult to meet the photo quality requirements using the same collection parameters in a complex outdoor environment. In addition, the quality of the images collected is significantly affected by outdoor conditions; hence, the following four factors need to be considered simultaneously when taking photos of tea chrysanthemums (as illustrated in **Figure 1**, the serial number of the images in the dataset is provided),

(1) Variety: Different varieties of tea chrysanthemums varies greatly in size, especially the size difference between the *Jinsihuangju* (**Figure 1A**) and *Hangbaiju* (**Figure 1B**). If the images are collected by the camera at the same distance, the number of flowers contained in one image will exhibit considerable diversity, further leading to unbalanced data which will affect the precision of the training models. Therefore, the distance to the flower should be adjusted between 30cm to 50cm according to the variety.
(2) Planting density: As the planting density of tea chrysanthemums is relatively high, which generally results in significant overlap (The cyan circle in **Figure 1**) and occlusion between flowers (The blue circle in **Figure 1**), can occur when the images are collected in the fixed angle, which can reduce the precision of detection models. To avoid these problems, three camera views, including ∼0°, ∼45°, and ∼90° , are chosen for image collection in the field.
(3) Field conditions: Tea chrysanthemums are planted in different regions under diverse field conditions. For *Jinsihuangju*, *Wuyuanhuangju*, and *Gongju*, a set of ropes and bamboo poles are used on both sides of tea chrysanthemums plants to avoid lodging. However, both bamboo poles (**Figure 1A**) and ropes (**Figure 1D**) in the images will influence the detection precision, which can also be solved by taking photos in the above three views.
(4) Photographing conditions: A large number of occlusions, overlap, direct light (The green circle in **Figure 1**), shadow (the red circle in **Figure 1**), and backlight (The orange circle in **Figure 1**) images are collected under the photographing conditions, such as wind and the light intensity changes. To reduce these external factor influences on image quality, a large number of images are necessary to detection models (Qi et al., 2022b).

There is currently no publicly available tea chrysanthemum dataset to the authors’ knowledge. Consequently, we provide an image dataset for six varieties of tea chrysanthemums in three camera view angles obtained under complex outdoor scenes, and this open-source image dataset can greatly promote the development of tea chrysanthemums detection methodology.

## 2 Value of the data

(1) RGB-based images of tea chrysanthemums in three view angles can provide sufficient flower features for detection models. This will further increase the detection precision while facing the overlap, occlusion, and shadow in complex outdoor scenes.
(2) Collecting large quantities of tea chrysanthemums images according to five different outdoor conditions, including occlusion, overlap, direct light, backlight, and shadow in complex outdoor scenes, are of great benefit for extending the applicability and enabling better precision of detection models.

## 3 Materials and methods

### 3.1 Collection and construction of the dataset

#### 3.1.1 Image acquisition

**Figure 2:**
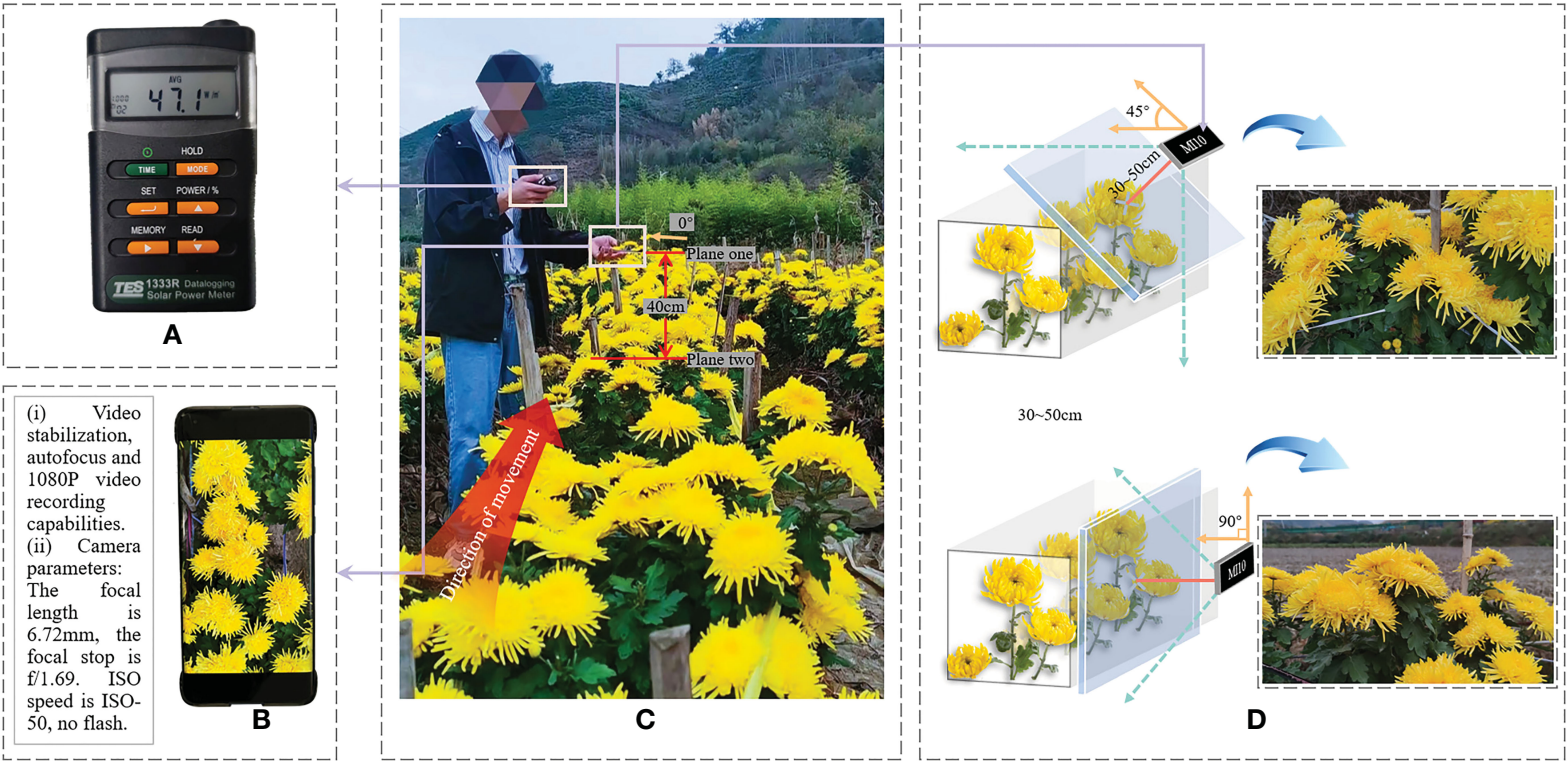
**(A)** TES-1333R Solar Power Meter, **(B)** Mi 10 phone, and **(C)** Image acquisition scene, **(D)** Schematic diagram of the camera angles.

With the continuous development of imaging technology, smartphones have become important media equipment for common image and video acquisition and their usage has higher flexibility. In this work, as illustrated in **Figure 2B**, the Mi 10 phone (Manufacturer: Xiaomi Corporation) is used for acquiring both images and videos, and more images can be extracted from the videos to enrich the image dataset. The videos are also included in the image dataset.

#### 3.1.2 Collection method

In **Figure 2**, the author took *Jinsihuangju* images at 4:00 p.m. in Xitou Village, She County, Huangshan City, Anhui Province, China. The TES-1333R Solar Power Meter (**Figure 2A**) in average mode was used to measure the average radiant illuminance before photography, describing the effect of lighting conditions on the photography, with the Solar Power Meter showing an average outdoor radiant illuminance of 47.1W/m^2^ at that time. Firstly, the Mi10 phone (**Figure 2B**) was held in the left hand and moved along the tea chrysanthemum (in the direction of the orange arrow in **Figure 2C**). Secondly, an angle of ∼0°, was selected for photography in **Figure 2C**, with a distance of 40cm between plane one, where the phone camera is located, and plane two, where the top canopy of the tea chrysanthemums is located. Since tea chrysanthemums heads are positioned differently, we used different angles for shooting that is better than a fixed angle. This will further acquire a large number of tea chrysanthemums images in different directions and reduce the noise from occlusion and overlap.

Therefore, to better display the complexity of the actual outdoor scenes, we used the following three angles, and the angles are not strictly fixed to fit the different orientations of photography.

•∼0°: The plane one, where the Mi 10 phone is located, is parallel to plane two, where the top canopy of the tea chrysanthemums is located, with an angle of ∼0° above the horizontal of the tea chrysanthemum.
•∼45°: The plane one, where the Mi 10 phone is located, is parallel to plane two, where the oblique side canopy of the tea chrysanthemums is located, with an angle of ∼45° above the horizontal of the tea chrysanthemum.
•∼90°: The plane one, where the Mi 10 phone is located, is parallel to plane two, where the lateral canopy of the tea chrysanthemums is located, with an angle of ∼90° above the horizontal of the tea chrysanthemum.

In addition, depending on whether the tea chrysanthemum planting density is high, it is necessary to choose another angle and shoot again for the same plants.

### 3.2 Image annotation and dataset production

As depicted in **Table 1**, the data on six varieties of tea chrysanthemums are collected in the form of images and videos, better displaying the complexity of the outdoor scenes. Taking the *Bo-chrysanthemum* as an example, the 221 images and videos with a total duration of 67 min were collected at Yaowang Village on 24 September 2022. Then FFmpeg (Get URL: https://ffmpeg.org/) was used to capture an image every 20 frames of the above video, and the resolution of all the captured images and the 221 images were adjusted into 1080x1920, all of which were stored in JPG format. Finally, the above-processed images were selected manually to delete the obscure images, leaving 11592 *Bo-chrysanthemum* images in the dataset, of which 221 images were reserved after the above processing step.

**Table 1:** Collection details about tea chrysanthemum dataset. 0.7.

Tea chrysanthemum	Time	Position	Total video duration	The final number of image (original images)
*Bo-chrysanthemum*	2022-9-24 7:00 9:00 11:00 14:00	Yaowang Village, Shibali Town, Qiaocheng District,Bozhou City, Anhui Province, China	67min	11592(221)
*Chuju*	2022-11-10 8:00 9:00 11:00 13:00	Chrysanthemum Expo Park, Nanqiao District,Chuzhou City, Anhui Province, China	84min55s	15275(25)
*Gongju*	2022-10-29 9:00 11:00 14:00 16:00	Xipo Village, She County, Huangshan City, Anhui Province, China	81min39s	14378(7)
*Hangbaiju*	2022-10-25 9:00 10:00 12:00 14:00	Minfeng Village, Shimen Town, Tongxiang City,Jiaxing City, Zhejiang Province, China	88min42s	16075(177)
*Jinsihuangju*	2022-10-28 9:00 11:00 14:00 16:00	Xipo Village, She County,Huangshan City, Anhui Province, China	81min39s	12519(13)
*Wuyuanhuangju*	2022-10-27 8:00 10:00 13:00 15:00	Xitou Village, She County,Huangshan City, Anhui Province, China	69min21s	11428(10)

Qi et al. (2022b) showed that up to 3000 images are sufficient to train and achieve better detection precision in the TC-YOLO model. Thus, in the dataset, the number of labelled images for each type of tea chrysanthemum was limited to 3000. The labelling software used was LabelImg (Get URL: https://github.com/heartexlabs/labelImg), which was used to annotate six tea chrysanthemums, labelling 18,000 images in total and saving the result of each image as an XML file.

In summary, we present an image dataset of six types of tea chrysanthemums (*Bo-chrysanthemum*, *Hangbaiju*, *Jinsihuangju*, *Wuyuanhuangju*, *Gongju*, and *Chuju*), a total of 81,276 images (1080×1920 pixels), captured using Mi10 phone. The image dataset was collected under five difficult-to-identify complex outdoor conditions: (1) direct light, (2) backlight, (3) shadow, (4) occlusion, and (5) overlap. Besides, this dataset also provides 453 original images (5760×3240 pixels) and videos (1080P and 60FPS) of tea chrysanthemums, which enables other researchers to use these datasets for further image analyses.

## 4 Direct link to deposited data and information to users

Publicly available datasets were contributed in this study. This data can be found at: https://dx.doi.org/10.21227/vc18-rv06.

## Data availability statement

The original contributions presented in the study are included in the article/supplementary material. Further inquiries can be directed to the corresponding author.

## Author contributions

SZ, LS, RH, ZG, and XW designed the research. SZ conducted the experiment. SZ and KH analyzed the data. SZ, KH, LS, and RV wrote the paper and revised it. SZ, JB, YZ, and YC labeled the data based on the images. All authors contributed to the article and approved the submitted version.
